# Nocardia masquerading as pulmonary malignancy in a patient with adult-onset immunodeficiency on ^18^F-FDG PET/CT

**DOI:** 10.1016/j.radcr.2024.07.058

**Published:** 2024-08-03

**Authors:** Harshil Dharamdasani Detaram, Phuong H.D. Nguyen, Veronica C. Wong, Han Loh, Robert Mansberg

**Affiliations:** a Nuclear Medicine and PET, Nepean Hospital, Penrith; b Sydney Medical School, The University of Sydney, Sydney

**Keywords:** Nocardia, Pulmonary malignancy, Adult onset immunodeficiency, FDG PET/CT

## Abstract

A 77-year-old man with a history of left nephrectomy for renal cell carcinoma and partial hepatectomy for cholangiocarcinoma underwent ^18^F-FDG PET/CT for assessment of an irregular lung lesion. FDG-PET demonstrated development of an intensely avid spiculated left lower lobe pulmonary lesion and intensely avid left pulmonary hilar nodes, raising suspicion for a malignancy. Eleven days following the PET study, the patient was admitted to hospital with an altered mental state. CT brain revealed diffuse round hyperdensities within the brain parenchyma. Microbiology of the lung lesion was positive for Nocardia Beijingensis and he was subsequently diagnosed with disseminated nocardiosis.

## Introduction

We describe a case of patient with multiple prior malignancies and ^18^F-FDG PET/CT findings concerning for new pulmonary neoplastic involvement, who was subsequently diagnosed with a systemic Nocardia Beijingensis infection. Nocardiosis presenting as a lung mass has been described previously on CT imaging [1], however, this case further demonstrates concurrent ^18^F-FDG PET/CT findings and a pattern of cerebral spread with the temporal acuity of systemic opportunistic infection in an immunocompromised host documented on imaging. This case highlights the importance of considering infection as a differential for lung masses, even with suspicion of metastatic spread, with ^18^F-FDG PET/CT findings potentially mimicking primary lung malignancy in the case of mass-forming nocardiosis.

## Case report

**Fig. 1:**
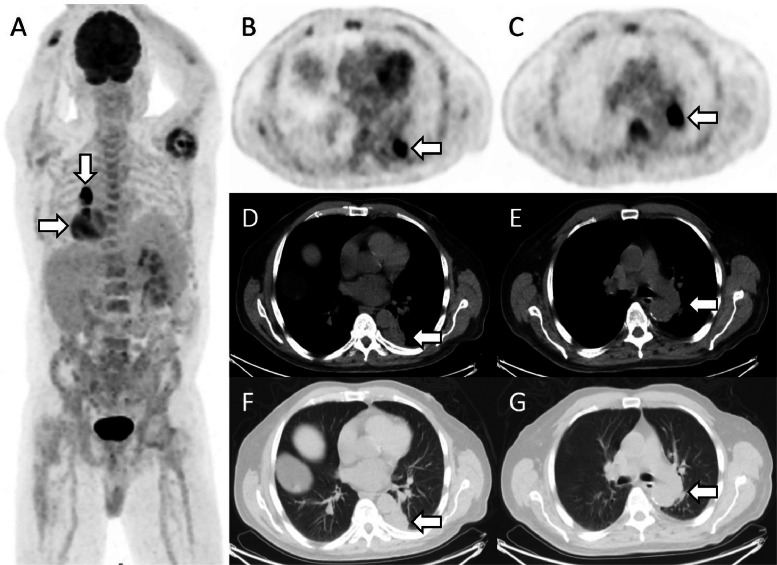
^18^F-FDG PET/CT maximal intensity projection image and CT images at presentation.

A 77-year-old man with a history of left nephrectomy for renal cell carcinoma and partial hepatectomy for cholangiocarcinoma underwent ^18^F-FDG PET/CT for assessment of an irregular lung lesion. The patient has a medical background including of nonalcoholic steatohepatitis cirrhosis with splenomegaly and thrombocytopenia and Paget's disease. The maximal intensity projection (Fig. 1) image (**A**) revealed 2 intensely FDG-avid lesions in the left lower lobe of the lung (SUVmax, 9.5) (**B**) and the left pulmonary hilum (SUVmax, 31.1) (**C**). Transaxial images from a concurrent diagnostic CT revealed an irregular lesion in the medial basal segment of the left lower lobe abutting the posteromedial pleura (arrowed) with adjacent pleural reaction (**D, F**) and enlarged left perihilar lymph nodes (arrowed) (**E, G**) suspicious for intensely avid malignancy in the left lower lobe pulmonary region with suspected nodal metastasis in the left pulmonary perihilar region.

**Fig. 2:**
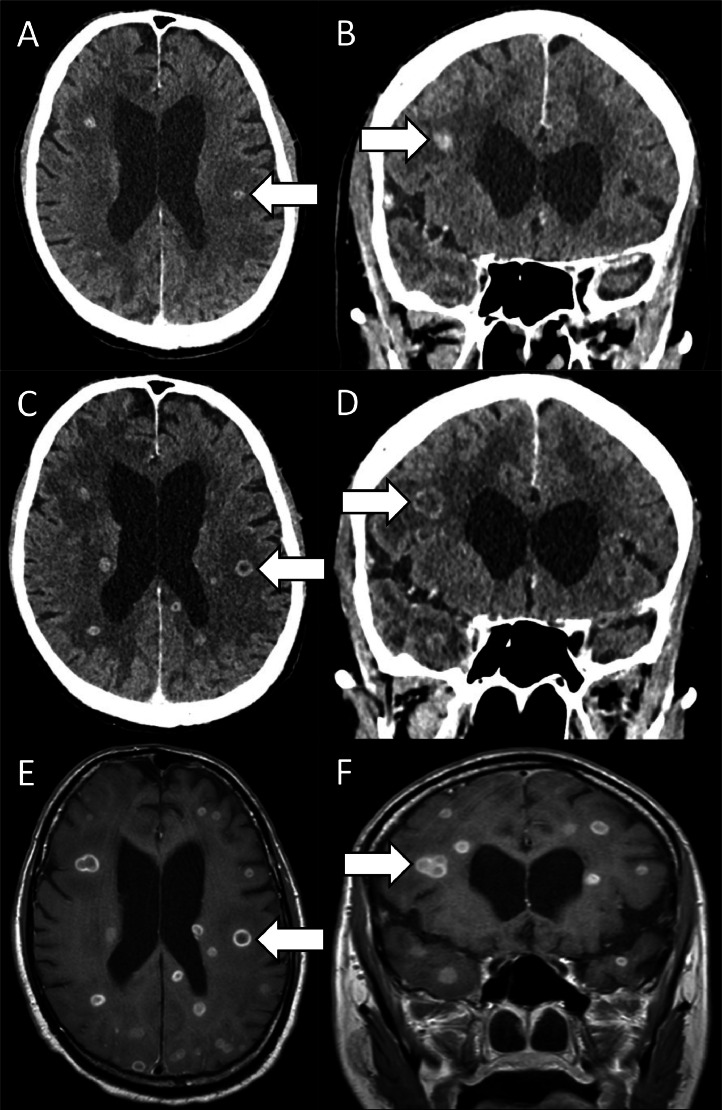
A contrast-enhanced CT and MRI of the brain at onset of neurological symptoms.

Eleven days following the PET study and prior to any intervention, the patient was admitted to hospital following a collapse with altered mental state. A contrast-enhanced CT of the brain (Fig. 2) revealed multiple round rim-enhancing lesions (arrowed) in the cerebrum, cerebellum and brainstem with surrounding vasogenic oedema (**A, B**). A month later the patient had a progress contrast-enhanced CT which revealed interval progression in size and quantity of the lesions (arrowed) (**C**)(**D**). On MRI T1-weighted postcontrast (gadobutrol [Gadovist 1.0]) images, the lesions (arrowed) similarly demonstrated ring enhancement with internal hypointensity (**E, F**). These findings were thought to be suspicious for development and progression of intracranial metastatic disease. However, core biopsy of the lung lesion shown in Fig. 1 demonstrated interstitial chronic inflammation and possible lipoid pneumonia with no evidence of malignancy. Microbiology of the lung biopsy was positive for Nocardia Beijingensis and he was subsequently diagnosed with disseminated nocardiosis involving the lung and brain. The patient was subsequently tested for anti-GM-CSF antibodies, which revealed a positive result. Which is theorized to have caused impaired pulmonary immunity due to pulmonary alveolar proteinosis resulting in susceptibility to pulmonary nocardiosis. Patient was started on empiric treatment of Bactrim (trimethoprim and sulfamethoxazole) and Meropenem.

**Fig. 3:**
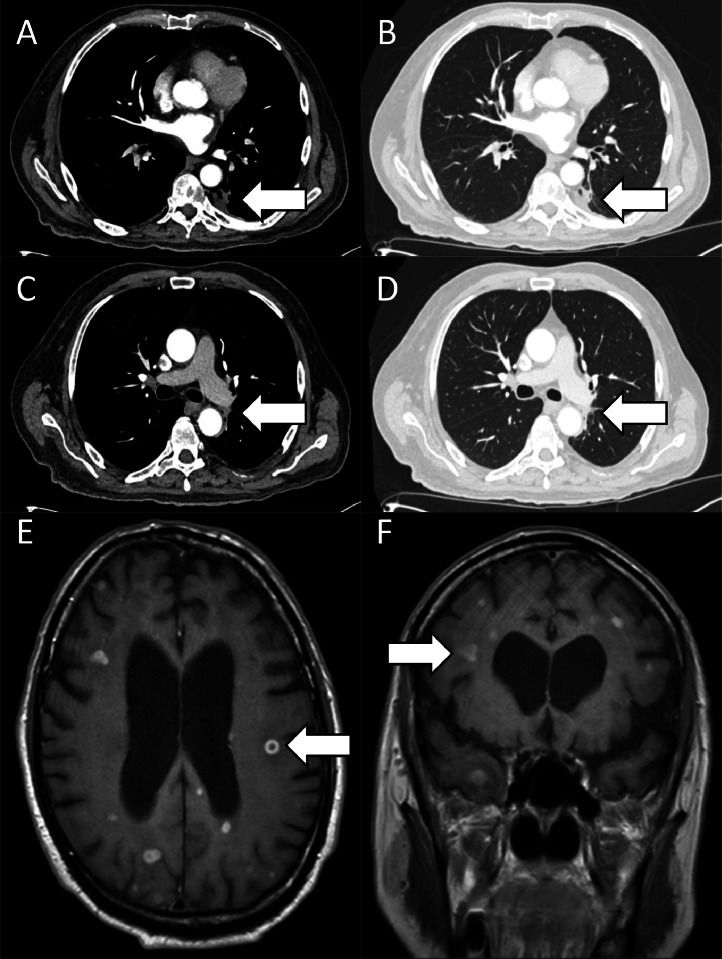
Contrast-enhanced axial CT images of the chest and contrast-enhanced MRI images of the brain taken a month after initiation of treatment.

Fig. 3 shows contrast-enhanced axial CT images of the left lower lobe lung lesion (arrowed) (**A, B**) and left perihilar pulmonary lymph nodes (arrowed) (**C, D**) taken a month after initiating systemic treatment for disseminated nocardiosis reveal interval reduction in size. On MRI T1-weighted postcontrast (gadobutrol [Gadovist 1.0]) images there has similarly been an interval reduction in size, number, and associated perilesional oedema of the diffusely distributed intra-axial rim-enhancing lesions (arrowed) (**E, F**).

## Discussion

Nocardia is a gram-positive bacillus typically manifesting as an opportunistic infection in immunocompromised hosts, with the bacteria residing in standing water, decaying plants and soil [2,3]. Nocardiosis can reveal the presence of adult-onset immunodeficiency due to autoantibodies against granulocyte colony stimulating factor resulting in autoimmune pulmonary alveolar proteinosis leading to pulmonary insufficiency and increased susceptibility to infection [4,5]. Disseminated nocardiosis often arises following pulmonary involvement and delay in diagnosis is implicated in treatment failure and poor prognosis [6]. Differential diagnosis for pulmonary nocardiosis would include other causes of airspace opacities, including infections, auto-immune conditions such as vasculitis and malignancy. Nocardiosis is an important differential in patients with lung masses and suspected infection.

Pulmonary findings can vary significantly, including presentations of irregular nodules, diffuse pulmonary infiltrates, lung abscesses, or pleural effusions [7,8]. The most common CT findings in pulmonary nocardiosis are nodules/masses with or without cavitation [9]. Additionally, rim-enhancing lesions were found to be the main radiological feature in patients with cerebral nocardiosis [10]. ^18^F-FDG PET/CT plays an important part in diagnosis and management. However, ^18^F-FDG is a glucose analogue and thus is used to evaluate a wide range of solid and hematological malignancies due to increased glucose utilization by malignant cells [11]. Infectious and inflammatory conditions also display ^18^F-FDG avidity through a shared mechanism and can therefore be misinterpreted as metastatic disease [11]. It is the role of the radiologist to refine the differentials by contrasting various imaging modalities. ^18^F-FDG PET/CT can guide biopsy by revealing the most metabolically active lesion and determine response to treatment as a recent study determined there may be a trend between CRP and maximum specific uptake values [12,13].

Treatment of disseminated nocardiosis typically involves long durations of antibiotic therapy ranging from 6 to 12 months, however, recent data has shown shorter durations of therapy can achieve similar results [14]. Empiric antibiotic therapy includes Bactrim (trimethoprim-sulfamethoxazole) 320 + 1600 mg 12-hourly plus either imipenem 500 mg IV 6-hourly, meropenem 2 g IV 8-hourly or amikacin 15 mg/kg IV once a day [15]. This is an important consideration given that patients can often have adverse reactions to treatment and extended antibiotic use results in increasing rates of highly resistant organisms. Incorporating ^18^F-FDG PET/CT at specific intervals from diagnosis may allow for better titration of treatment duration [12].

## Conclusion

This case of temporally documented progressive disseminated Nocardiosis mimicking a primary pulmonary malignancy with concurrent ^18^F-FDG PET/CT and CT findings highlights the importance of a maintaining a wide range of differentials when considering an undifferentiated lung mass with suspected metastatic spread. Additionally, ^18^F-FDG PET/CT is valuable in guiding biopsy and may have an ongoing role in determining treatment response.

## Patient consent

This is to confirm that informed consent was obtained by the authors from the patient for this case report to be published.
